# John Langdon Haydon Down (1828–1896)

**DOI:** 10.1007/s00415-021-10601-x

**Published:** 2021-05-11

**Authors:** Andrzej Grzybowski, Joanna Żołnierz

**Affiliations:** 1 Department of Ophthalmology, University of Warmia and Mazury, Olsztyn, Poland; 2 Institute for Research in Ophthalmology, Gorczyczewskiego 2/3, 61-553 Poznan, Poland; 3 Interfaculty Center for Didactics, Medical University of Lublin, Lublin, Poland

**Fig. 1:**
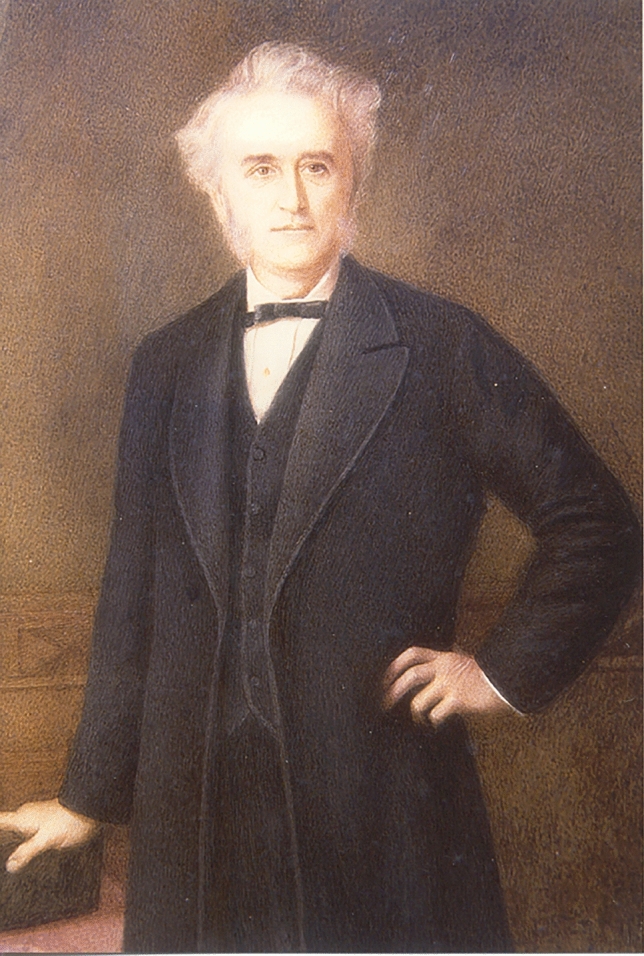
John Langdon Haydon Down (1828–1896). Reproduced by permission of the Langdon Down Museum of Learning Disability and the Down’s Syndrome Association UK

John Langdon Haydon Down was born on November 18, 1828, at Torpoint, a working-class village in Cornwall (Fig. 1) [1, 2]. Down was raised in a religious family and was the youngest of six children [2, 3]. His father, Thomas Joseph Almond Down, was a grocer, linen trader, pharmacist, and presumably addicted to alcohol. He bankrupted the family business three times, and when John was 14, he took him away from school to help run a shop [3]. In the father’s shop, Langdon Down spent the next 4 years [2].

Langdon Down’s medical career began with his apprenticeship under a London surgeon. These first experiences made the young Down aware that he had too little knowledge and skills to practice medicine. So he decided to attend one of the best courses in London, organised by the Royal Pharmaceutical Society. Langdon Down established himself as a brilliant student by passing two professional exams in one year. However, he did not plan a pharmacist’s career, so he returned to his hometown and, used his knowledge to produce pharmaceuticals, thus significantly improving the financial situation of the family store. Soon afterwards, the Royal Pharmaceutical Society offered him a job as a laboratory assistant. However, Down did not work long in this position because he fell ill, most likely with tuberculosis, and returned to Torpoint, where he slowly recovered [2].

In 1853, after his father’s death, John returned to his career plans and began studies in the London Hospital Medical School [2, 3]. Here, he was also recognised as a talented and intelligent student. In the senior year of his studies, he was awarded a gold medal in three subjects: surgery, medicine and obstetrics, as the best student of his class [2, 3]. In poverty, Down had the opportunity to live in London at no cost with his sister and her husband, Philip Crellin. That was how he met his future wife, Mary Crellin, who was Philip’s sister. The couple married in 1860 [4]. After passing the qualifying exams of the Royal College of Surgeons and the Worshipful Society of Apothecaries, he began working as a resident accoucheur–obstetrician [2, 3]. Although that was an unpaid job, he was guaranteed food and accommodation, which created favourable conditions for working for and passing his London MB in 1858 [2, 3]. In the same year, Down, with a view to his unfavourable financial situation and the plan of future marriage, decided to accept the job offer of a medical superintendent at the Royal Earlswood Asylum for Idiots in Redhill [3]. Down worked at Earlswood for ten years and from 1859 simultaneously as an assistant physician at the London Hospital [1, 3].

The Royal Earlswood Asylum for Idiots, one of the most prominent institutions for people with mental disorders, had been criticised by the Commissioners in Lunacy [2–4]. Down, under the direction of John Conolly, helped restore the institution’s good reputation through administrative changes and by providing a proper care. Particular attention was paid to the excellent nutrition and professional training, and physical punishment was forbidden. This style of managing the asylum by Down was influenced not only by the thought of John Connolly but also by Édouard Séguin’s concept of “moral treatment”. Séguin’s theory, emphasising the role of motor training and non-use of physical restraint, also strongly influenced the vision of Normansfield House, founded in later years by Down [2].

Fascinated by the work of John Conolly in the field of ethnology and in anthropological research, popular at the time, Down decided to classify the disorders observed in the young patients at Earlswood. Referring to Blumenbach’s “degenerative hypothesis” of racial origins, Down began his research by measuring the diameter of the heads and describing the facial features [2]. The documentation was based on photography taken by Down (about 200 of which survive to this day), in what was the greatest clinical documentation from the Victorian era [2, 3]. In 1866, Down presented his research in an article in which he described a “Mongolian type” [5].

In 1868, Down resigned from The Royal Earlswood Asylum. The reason for the resignation was a deterioration of contacts with the Lords of Earlswood, who first objected to the remuneration of wages to Down’s wife for her work in the asylum and then refused to finance an exhibition of Earlswood patients’ works in Paris [4]. This decision allowed Down to return to his full-time job at a London hospital and set up his own private home for those with developmental and intellectual disabilities at Normansfield [3, 4]. In the Normansfield House, smoking was forbidden, and particular attention in caring for the residents was paid to the correct articulation and training of the facial muscles and personal hygiene [2]. The institution had a good reputation, as evidenced by the increase in the number of residents of the House—in 1868, when the House was established, 19 children lived in Normansfield, and in 1896 their number had grown to 160 [3, 4].

In the book published in 1887, Down presented a very detailed description of the characteristics of the “mongoloid idiot” [4, 6]. Furthermore, that same year, at the invitation of the Medical Society of London, he gave 3 Lettsomian Lectures [8]. After their publication, the scientific community often referred to Down’s ethnic classification [2]. In addition to significant discoveries about the condition, today called in his honour Down syndrome, John Langdon was the first to publish a description of the Prader–Willi syndrome [2, 7].

John Langdon Down died suddenly in 1896 at the age of 67 years. On the day of his funeral, all of Hampton Wick mourned, crowds of passers-by stood in silence, honouring the excellent physician and philanthropist. Down’s body was cremated, and his ashes kept at Normansfield. They were scattered after his wife’s death, along with her ashes [4]. Down’s descendants ran Normansfield until 1951 when the NHS took it over [3].

On behalf of all authors, the corresponding author states that there is no conflict of interest.
